# Influence of ZnO Nanoparticles on the Color and Surface Roughness of Composite and Glass-Ionomer Materials

**DOI:** 10.3390/jfb17070343

**Published:** 2026-07-15

**Authors:** Sanja Ilić, Neda Ninkovic, Branislav Sredanovic, Goran Vučić, Ljiljana Božić, Ljubica Škrbić, Jovana Kuzmanovic Pficer, Dragica Manojlovic

**Affiliations:** 1 Dental Clinic, Faculty of Medicine, University of Banja Luka, Bulevar vojvode Petra Bojovića 1a, 78000 Banja Luka, Bosnia and Herzegovina; sanja.ilic@med.unibl.org (S.I.); ljubica.skrbic@med.unibl.org (L.Š.); 2 Department of Restorative Odontology and Endodontics, School of Dental Medicine, University of Belgrade, Rankeova 4, 11000 Belgrade, Serbia; neda.nikolic@stomf.bg.ac.rs; 3 Department for Production and Computer Aided Technologies, Faculty of Mechanical Engineering, University of Banja Luka, Bulevar vojvode Stepe Stepanovića 71, 78000 Banja Luka, Bosnia and Herzegovina; branislav.sredanovic@mf.unibl.org; 4 Faculty of Technology, University of Banja Luka, Bulevar vojvode Stepe Stepanovića 73, 78000 Banja Luka, Bosnia and Herzegovina; goran.vucic@tf.unibl.org; 5 Department of Microbiology and Immunology, Faculty of Medicine, University of Banja Luka, Save Mrkalja 14, 78000 Banja Luka, Bosnia and Herzegovina; ljiljana.bozic@med.unibl.org; 6 Department for Medical Statistics and Informatics, School of Dental Medicine, University of Belgrade, Dr. Subotica 1, 11000 Belgrade, Serbia; jovana.kuzmanovic@stomf.bg.ac.rs

**Keywords:** zinc oxide nanoparticles, composite resins, glass ionomer cements, color stability, surface roughness

## Abstract

Objective: The effect of incorporating various concentrations of 210 nm ZnO nanoparticles (ZnO-NPs) into resin-based composites (RBCs) and glass-ionomer cement (GIC) on their color and surface roughness after immersion in red wine is evaluated. Materials and Methods: 12 experimental groups (*n* = 12) were tested, including three commercial restorative materials: a microhybrid RBC (Gradia Direct^®^), a nanohybrid RBC (Evetric^®^) and a GIC (Fuji IX^®^). These were modified with ZnO-NPs at weight concentrations of 1%, 2%, and 3%. Color coordinates (L*,a*,b*) were measured using a spectrophotometer (CIEDE2000 formula) before and after 24 h immersion in red wine (37 °C). Surface roughness (Ra) was analyzed using contact profilometry. Results: The addition of ZnO-NPs increased the lightness (L*) and decreased the a* values in all materials. In resin-based composites, even a 1% concentration of ZnO-NPs caused a color change above the clinical acceptability threshold (ΔE_00_ > 1.8; observed values ranged from 3.2 to 8.0). After immersion in wine, all materials exhibited further discoloration (ΔE_00_ up to 12.5 for composites). Surface roughness values increased from 0.42 μm (control composite) to 1.95 μm (3% ZnO-NP-modified GIC). Conclusions: Addition of ZnO-NPs influences the aesthetics and topography of restorative materials. Their concentration must be balanced to maintain visual and surface integrity.

## 1. Introduction

Over the years, resin-based composites (RBCs) have been significantly improved through the development and modification of fillers and polymer matrix to achieve outstanding aesthetic and favorable mechanical properties, as well as reduced contraction [1,2]. In recent years, considerable research efforts have been directed towards developing materials with potential antimicrobial effects to enhance the clinical longevity of restorations and reduce the risk of secondary caries [3]. Compared to other materials and hard dental tissues, RBCs show a greater tendency for biofilm formation, which increases the risk of demineralization and secondary caries. Their composition typically does not include natural antibacterial components, and some bacteria can even metabolize resin ingredients [4,5]. On the other hand, glass-ionomer cements (GICs), despite their chemical bonding to tooth tissue and fluoride release, possess certain drawbacks; therefore, efforts are being made toward their optimization to improve clinical performance in terms of reducing surface roughness and enhancing biological activity, mechanical properties and antimicrobial effects [6,7].

Earlier approaches to achieving antimicrobial properties included the incorporation of agents such as fluoride and chlorhexidine, or chemical modification of the resin matrix with quaternary ammonium compounds. These approaches can be limited by long-term efficacy or potential disruption of the material’s mechanical properties [8,9,10]. An increasingly researched approach involves incorporating metal and metal-oxide nanoparticles into restorative materials. Silver, gold, and zinc are among the most frequently utilized metals with established antibacterial properties [8,11,12]. Zinc oxide nanoparticles (ZnO-NPs) attract particular attention due to their pronounced antibacterial effect, biocompatibility and chemical stability [12,13,14]. Numerous studies reported that the incorporation of ZnO-NPs effectively inhibits the growth of cariogenic microorganisms; this antimicrobial activity is primarily attributed to the gradual release of Zn^2+^ ions, the generation of reactive oxygen species (ROS) and the disruption of bacterial cell membrane integrity. However, enhancing the antimicrobial activity of these materials without compromising their mechanical and aesthetic properties or surface characteristics remains a significant challenge [15].

The alignment of optical properties with dental tissue, as well as their stability over time are key parameters for successful restorations from the perspective of aesthetic acceptability. Initial aesthetic properties depend on the material composition, degree of polymerization, and surface finishing [16]. Unlike silver nanoparticles (Ag-NPs), which compromise color even at minimal concentrations, ZnO-NPs have often been considered less likely to compromise aesthetic properties due to their white color [17,18]. However, even low concentrations can induce color changes that exceed clinical acceptability thresholds, particularly in resin-based composites. This highlights the need for careful optimization of ZnO-NP content and dispersion strategies to maintain both antimicrobial and aesthetic performance. Color stability over time represents another critical factor for the long-term clinical success of aesthetic restorations. Color changes in restorations can generally result from extrinsic factors, such as pigments from food, beverages, or smoking, as well as intrinsic chemical processes involving water absorption and degradation [19,20,21]. Mechanical wear, microleakage, and surface roughness can further influence color changes. Added ZnO-NPs could potentially affect the material’s roughness and accelerate staining if they are not chemically stable or properly surface-modified [22].

The roughness of the restoration is influenced by the characteristics of the filler and resin matrix within the material composition, as well as the finishing and polishing of the restoration. Higher surface roughness also affects light reflection and visual gloss [23,24] and may influence staining susceptibility, as rougher surfaces facilitate pigment and plaque accumulation [25]. Added ZnO-NPs, due to their small particle size, could contribute to lower surface roughness if the particle dispersion within the organic matrix is uniform [26,27]. Additionally, the effect on reduced plaque adhesion could indirectly contribute to preserving the restoration’s color. On the other hand, inadequate concentration and agglomeration of ZnO-NPs can lead to an increase in surface roughness and the disruption of aesthetic stability [12]. To achieve a balance between enhancing antimicrobial activity and preserving optical properties and long-term color stability, it is necessary to carefully optimize the concentration, particle size, and dispersion.

Recent studies have investigated the incorporation of ZnO-NPs into dental cements and composites to improve mechanical strength, antibacterial activity, and biostability [13,28,29]. Notably, Jowkar et al. [26] and Malekhoseini et al. [29] explored the effects of ZnO-NPs addition on resin-modified glass ionomer cements, focusing primarily on mechanical properties such as microhardness and surface roughness, as well as antibacterial potential [26,29]. While these studies have contributed important insights, they predominantly examined a single class of restorative material and did not systematically address the influence of ZnO-NPs on key optical properties, such as initial color, color stability, and clinically acceptable color thresholds. Furthermore, there remains a lack of comparative data across multiple restorative material types and under conditions that mimic real-world clinical challenges, such as exposure to common staining agents. The present study is novel in providing a direct, side-by-side evaluation of how ZnO-NPs influence the color and surface roughness of both resin-based composites (microhybrid and nanohybrid) and conventional glass-ionomer cement. This comprehensive approach offers new insights into possible material-specific effects and guides the future optimization of nanoparticle-modified restorative materials. The present study provides:

- A comparative analysis of the effects of ZnO-NP incorporation into both resin-based composites (RBCs) (microhybrid and nanohybrid) and conventional glass ionomer cement (GIC);
- A systematic evaluation of not only surface roughness but also initial color (L*, a*, b*) and color stability following exposure to a clinically relevant staining agent (red wine), interpreted using established clinical acceptability thresholds (ΔE_00_);
- Insights into the potential trade-offs between surface and aesthetic properties when ZnO-NPs are added to restorative materials, using commercially available products and clinically relevant experimental conditions.

By expanding the scope beyond previous studies and focusing on clinically meaningful optical and surface outcomes across different material classes, this work aims to provide a comprehensive and clinically applicable understanding of the implications of ZnO-NPs incorporation in restorative dentistry. The aim of this study was to investigate the influence of different concentrations of added ZnO-NPs in RBCs and GIC on initial color, color change after immersion in red wine and material surface roughness.

Null Hypotheses: There is no statistically significant difference in initial color and color stability between the tested commercial RBCs and GIC compared to the same materials after the incorporation of ZnO-NPs and after immersion in red wine, regardless of their concentration. There is no statistically significant difference in surface roughness between the tested commercial RBCs and GIC compared to the same materials after the incorporation of ZnO-NPs, regardless of their concentration.

## 2. Materials and Methods

### 2.1. Preparation of Materials

Twelve experimental groups (*n* = 12) were tested: 3 commercial materials (Gradia Direct^®^, (microhybrid RBC), GC Corporation, Tokyo, Japan, Evetric^®^ (nanohybrid RBC), Ivoclar Vivadent, Schaan, Liechtenstein and Fuji IX^®^ (GIC), GC Corporation, Tokyo, Japan) and 9 experimental materials obtained by modifying the above-mentioned materials with the addition of 1 wt%, 2 wt% or 3 wt% ZnO-NPs (Table 1). ZnO-NPs (~210 nm) were synthesized from ZnSO_4_ and NaOH and manually incorporated into the commercial materials according to ISO 4049:2019 standards [30]. The 1–3 wt% concentration range was selected based on previous studies demonstrating that these levels are sufficient to induce significant changes in material properties and are commonly used as a benchmark for preliminary evaluation [14]. Pilot tests further confirmed that concentrations above 3% resulted in excessive agglomeration and poor material handling; therefore, 3 wt% was determined as the maximum for this study.

For the synthesis of ZnO nanoparticles (ZnO-NPs), a 1 M aqueous solution of zinc sulfate hydrate (ZnSO_4_ · xH_2_O, analytical grade, Semikem, Sarajevo, Bosnia and Herzegovina) served as the Zn^2+^ precursor. Under continuous stirring at 70 °C on a magnetic stirrer, an equal volume of 2 M sodium hydroxide solution (NaOH, analytical grade, Lach-Ner, Neratovice, Czech Republic) was added dropwise. The resulting suspension was stirred for an additional 2 h at the same temperature and subsequently aged overnight at room temperature. The precipitated Zn(OH)_2_ was isolated by centrifugation, washed multiple times with distilled water and 96% ethanol (Semikem, Sarajevo, Bosnia and Herzegovina) to remove residual NaOH, and the obtained paste was calcined at 300 °C until a constant mass was achieved to ensure full conversion into ZnO. The calcined product was ground in an agate mortar to yield a ZnO nanopowder. The UV–Vis absorption spectrum of the aqueous ZnO-NP dispersion was recorded, and the average particle size was calculated from the absorption maximum (λ_max_ = 389 nm). The synthesized ZnO-NPs displayed a spherical morphology. This synthesis route was adopted from previous research [31].

Scanning electron microscopy (SEM) of the ZnO-NP powder was performed at magnifications of 7500×, 10,000× and 15,000×.

The crystalline structure of the ZnO-NP powder was analyzed at room temperature using an automated multipurpose X-ray diffractometer (XRD; Rigaku, Japan) with preinstalled SmartLab Guidance software v1.0 (Rigaku, Japan). Diffraction patterns were recorded over a 2θ range of 20–90° range, with a step size of 0.02° and a scanning speed of 10°/min.

ZnO-NPs were added to the tested commercial materials at weight fractions of 1 wt%, 2 wt% and 3 wt% relative to the base material. After weighing on an analytical balance, the nanoparticles were manually incorporated into the commercial materials and homogenized by hand mixing on a glass slab using a metal spatula for 1–3 min. This approach was selected for its accessibility and feasibility in preliminary investigations. Importantly, prior studies have also utilized manual or low-tech mixing methods when initially evaluating nanoparticle-modified dental materials, demonstrating that such an approach is acceptable for pilot or feasibility studies [22,32,33,34]. These studies found that while this method may not achieve ideal nanoparticle dispersion, it is practical and reproducible in laboratory settings, reflecting real-world limitations in clinical or small-scale laboratory practice. The prepared materials were stored in dark tubes to prevent premature polymerization. The homogeneity of nanoparticle distribution was evaluated using scanning electron microscopy (SEM; JEOL JSM-T220, Akishima, Japan). SEM imaging was performed on cured/hardened samples containing different concentrations of ZnO-NPs at different magnifications.

Polymerization of RBC samples was performed using a Bluephase G2 LED curing light (Ivoclar Vivadent, Schaan, Liechtenstein) according to the manufacturer’s instructions. GIC samples were allowed to set for 15 min at room temperature, in accordance with the ISO 4049:2019 standard [30].

### 2.2. Color Measurements

For color testing, disk-shaped samples were prepared with a diameter of 10 mm and a thickness of 2 mm. The top and bottom surfaces of the samples were in contact with glass slides covered with celluloid strips, ensuring uniform pressure and a fixed distance of 1 mm between the light source and the sample. To avoid undercuring, the curing scheme from the ISO 4049:2019 standard was used [30]. Briefly, a five-point irradiation pattern was applied to each sample: four at the 12, 3, 6, and 9 o’clock positions, followed by a final irradiation in the center. Immediately after polymerization (RBCs) and setting (GICs), the samples were carefully removed from the molds and polished using the Super-Snap Buff system (Shofu Dent Corp., Kyoto, Japan) in the following sequence: medium, soft, super-soft, and super-polished, for 10 s per disk in a moist environment. The polished samples were then immersed in 15 mL of distilled water and incubated in a water bath at 37 °C for 24 h, in compliance with the ISO 4049:2019 standard [30]. Afterwards, the samples were gently blot- dried with paper towels and the initial color (baseline) was measured. The samples were then immersed in red wine (12.5% alcohol) for 24 h at 37 °C to evaluate color stability. The 24 h continuous immersion in the staining solution is a widely used accelerated aging protocol in dental research, intended to approximate the cumulative staining effects of several months of regular beverage exposure in the oral environment [35]. This approach enables an efficient comparison of color stability and has been validated in previous studies examining the impact of staining agents on restorative materials.

Diffuse reflectance spectra were recorded using a Konica Minolta spectrophotometer (CM-2600d, Konica Minolta Sensing, Sakai, Japan) across a wavelength range of 360–740 nm with a 1 nm step. Three measurements were conducted per sample, and the mean values were calculated. Reflectance spectra were determined relative to a calibration white standard. The spectrophotometer was calibrated according to the manufacturer’s recommendations using BaSO_4_ as the white reference. To minimize background interference, all measurements were performed against a uniform white background. The measurement geometry was configurated to d/8° to ensure consistency and comparability with previous studies. Color coordinates (L*, a*, and b*) were subsequently calculated from the diffuse reflectance spectra under standard illuminat D65 using the CIEDE2000 formula [36]:

$${\Delta E}_{00}=\sqrt{{\left(\frac{{\Delta L}^{\prime }}{k_{L}S_{L}}\right)}^{2}+{\left(\frac{{\Delta C}^{\prime }}{k_{C}S_{C}}\right)}^{2}+{\left(\frac{{\Delta H}^{\prime }}{k_{H}S_{H}}\right)}^{2}+R_{T}\left(\frac{{\Delta C}^{\prime }}{k_{C}S_{C}}\right)\left(\frac{{\Delta H}^{\prime }}{k_{H}S_{H}}\right)},$$

where ΔL′, ΔC′ i ΔH′ represent the adjusted values of metric CIELAB differences in lightness, chromaticity, and hue; S_L_, S_C_ and S_H_ are weighting functions; k_L_, k_C_, and k_H_ are parametric factors; and RT is the chromaticity/hue interaction rotation term. ΔE_00_ values lower than 1.8 were considered clinically acceptable color changes [37].

### 2.3. Surface Roughness Measurement

Ten samples for each group (*n* = 10) were prepared in plastic molds with dimensions of 30 × 3.3 × 3 mm. Polymerization was carried out in accordance with the ISO 4049:2019 standard [30]. Each sample was polymerized at five points (left, right, top, bottom, and center), followed by a final exposure using circular motions. Immediately after polymerization/setting, the samples were removed from the molds, polished, and incubated in a water bath according to the protocol described in the previous experiment for color measurement. The surface roughness of the tested materials was analyzed via contact profilometry using Mitutoyo SJ-310 profilometer (Kawasaki, Japan). The primary parameter used to describe surface roughness is (Ra), which represents the arithmetic mean of the absolute deviations of the surface profile from the mean line within the given measurement length. Surface roughness (Ra) was measured after polishing and 24 h storage in distilled water. No additional roughness measurements were performed following red wine exposure in this study.

### 2.4. Statistical Analysis

Statistical analysis was performed using the commercial software package SPSS Statistics, version 22.0 (IBM Corp., Armonk, NY, USA). Both descriptive and inferential statistical methods were employed. Descriptive statistics for continuous variables were expressed as means with standard deviations (SD) or medians with interquartile ranges (IQR), depending on the distribution. The selection of statistical tests for analyzing the numerical characteristics of the observations was determined by the nature of the data distribution. For parametric datasets, one-way ANOVA followed by Bonferroni-adjusted post hoc comparisons was performed. For non-parametric datasets, Kruskal–Wallis tests followed by Mann–Whitney U tests with Holm correction were applied to adjust for multiple comparisons, with *p*-value adjustments for multiple comparisons performed via the Holm correction implemented in R software version 4.5.1.

Sample size was calculated based on pilot study data for the primary outcomes of color change and surface roughness. An a priori power analysis was performed using G*Power software (version 3.1.9.4; Heinrich Heine University, Düsseldorf, Germany) for the difference between two means. Effect sizes derived from the pilot study were 1.314 for color change and 1.263 for surface roughness. The significance level was set at α = 0.05, and the statistical power was set at 80%. The analysis indicated that six samples per group were required to detect significant differences for both outcomes.

## 3. Results

**Figure 1:**
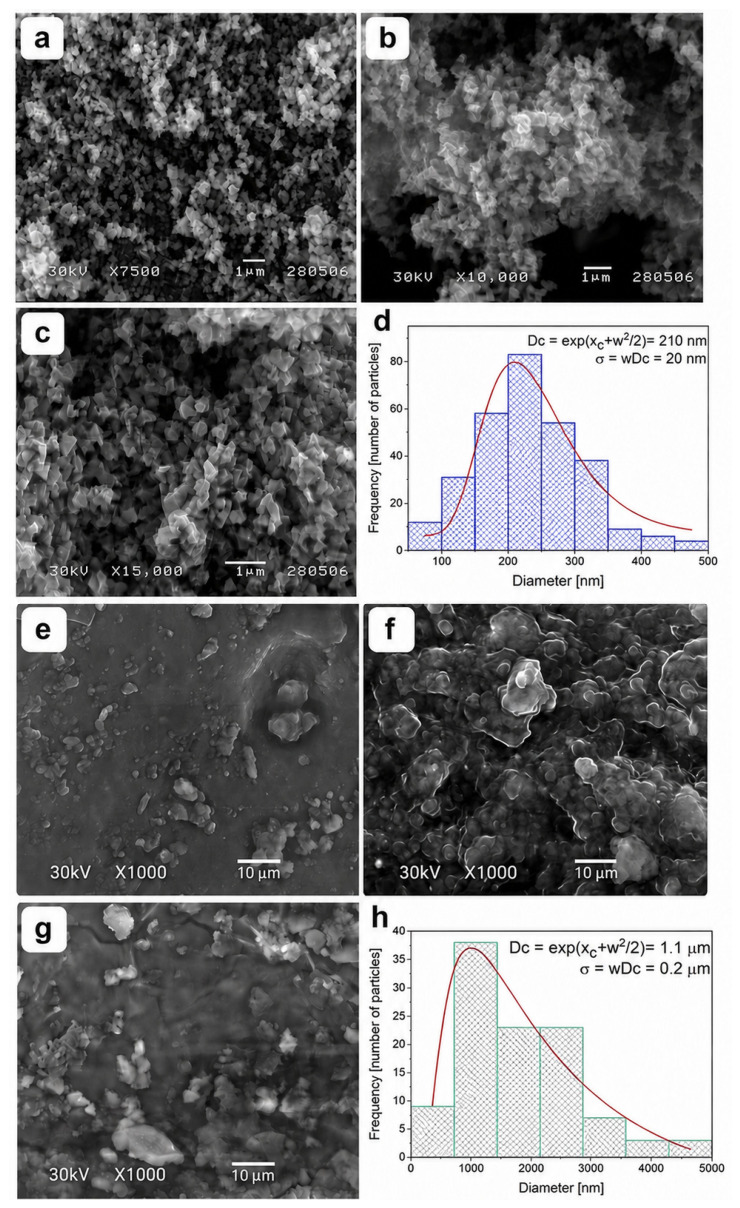
Scanning electron microscopy (SEM) images ZnO-NPs and restorative materials modified with ZnO-NPs. (**a**) Image of ZnO-NPs at 7500× magnification. (**b**) Image of ZnO-NPs at 10,000× magnification. (**c**) Image of ZnO-NPs at 15,000× magnification. (**d**) Size distribution of ZnO-NPs showing particles’ size of approximately 210 nm in diameter. (**e**) Gradia Direct with 2 wt% ZnO-NPs (Group G3) at 1000× magnification, showing regions of both uniform dispersion and visible particle agglomeration within the resin matrix. (**f**) Evetric with 1 wt% ZnO-NPs (Group E2) at 1000× magnification, illustrating the presence of ZnO-NPs distributed throughout the composite and occasional clusters. (**g**) Fuji IX with 1 wt% ZnO-NPs (Group F2) at 1000× magnification, highlighting the distribution pattern of nanoparticles in the glass ionomer cement and areas of particle aggregation. (**h**) Size distribution of ZnO-NPs in restorative materials showing particles’ size of approximately 1.1 μm in diameter.

Figure 1 illustrates the particle size distributions of the ZnO-NPs both prior to and following their incorporation into the materials, as determined via SEM analysis. The mean particle size was estimated from SEM images using ImageJ software version 1.54t, based on the analysis of 250 particles and subsequent log-normal fitting. Although the SEM micrographs reveal distinct zones of uniform particle distribution within the resin matrix, agglomerated particles are clearly observed rather than a monodispersed state.

**Figure 2:**
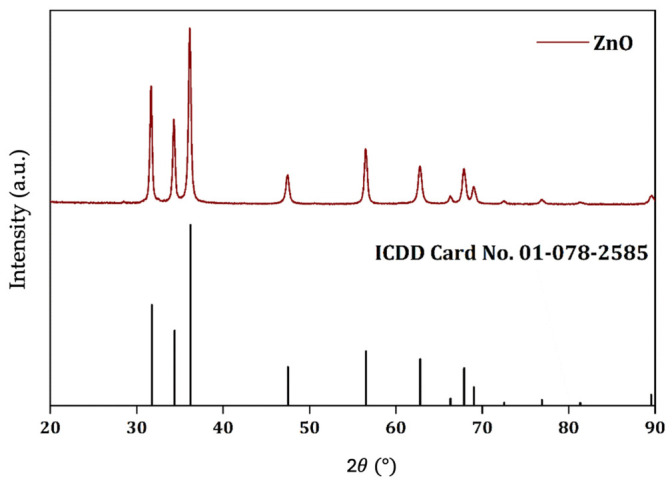
XRD pattern of the synthesized ZnO-NP sample.

Figure 2 displays the XRD pattern of the synthesized ZnO-NPs. The X-ray diffraction (XRD) analysis of the synthesized ZnO-NPs shows sharp diffraction peaks that index perfectly to a hexagonal structure within the *P*6_3_mc space group, consistent with ICDD card no. 01-078-2585.

The crystallite size of the sample was determined using the Scherrer equation applied to the three most intense diffraction peaks in the XRD pattern, corresponding to the (the (100), (002), and (101) *hkl* planes. Using the Cu-Kα radiation wavelength (λ=1.540 Å) and a shape factor K=0.89, the full width at half maximum (FWHM, β) and corresponding Bragg angles (θ) in radians were obtained for each peak. To improve the accuracy of the crystallite size calculation, a LaB_6_ standard was used for instrumental broadening correction ($\beta =\sqrt{\beta_{measured}^{2}-\beta_{instrumental}^{2}}$).

Applying the formula:

$$D=\frac{K\lambda }{\beta cos(2\theta /2)},$$

the calculated crystallite sizes were 28.2 nm, 26.5 nm, and 24.8 nm (Table 2). Therefore, on average, each particle consists of eight crystallites (Figure 2).

The obtained values for L*, a*, and b* color coordinates for all tested materials are shown in Table 3.

By analyzing the L*, a*, and b* values, it is observed that with the addition of ZnO-NPs, the materials become brighter (increase in L*). The change in brightness is more pronounced in both composite materials compared to the glass ionomer cement. In all three materials, a decrease in the a* parameter values (meaning less red and moving towards green) was observed after the addition of ZnO-NPs. While the b* values decreased in both composite materials (less yellow), an increase was observed in Fuji IX (moving more towards yellow).

**Figure 3:**
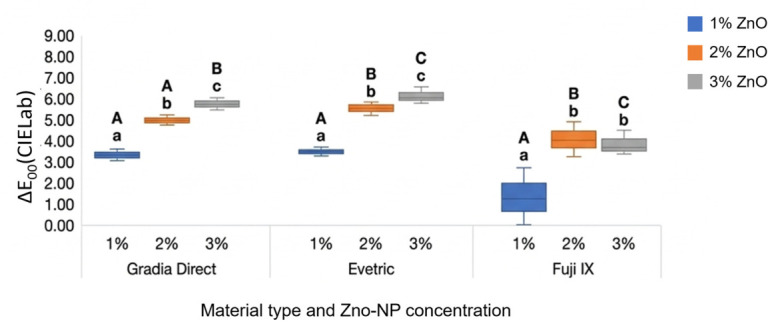
Changes in the initial color of the materials (ΔE_00_) after the addition of different concentrations of ZnO-NPs. Same uppercase letters indicate no significant differences between different materials with the same ZnO-NP concentration (*p* > 0.05); same lowercase letters indicate no significant differences within one type of material with different ZnO-NP concentrations (*p* > 0.05). Uppercase letters above the bars indicate no statistically significant differences between different materials with the same ZnO-NPs concentration (*p* > 0.05). Lowercase letters indicate no statistically significant differences within one type of material across different ZnO-NP concentrations (*p* > 0.05). Different letters (either uppercase or lowercase) denote statistically significant differences (*p* < 0.05) between the corresponding groups. The labels “A”, “B”, “C”, etc., are assigned based on the values of the parameter. “A” is assigned to the material with the highest value. The next material is assigned label “B” if the difference is statistically significant; otherwise, it is labeled “A”. The following material is compared with the previous one using the same principle. Lowercase letters within each material are assigned according to the same principle as uppercase letters.

The results of the initial color change (ΔE_00_) of the materials after adding different concentrations of ZnO-NPs are shown in Figure 3.

For Fuji IX, statistically significantly lower ΔE_00_ values (*p* = 0.000) were recorded for all tested concentrations of added ZnO-NPs compared to RBCs. The greatest change in initial color was observed in Evetric groups for all tested concentrations of added ZnO-NPs, although the difference compared to Gradia Direct was not statistically significant at 1% ZnO-NPs (*p* = 0.288). In RBCs groups, even a low concentration (1 wt%) of ZnO-NPs (G2 and E2 groups) led to a color change above the threshold of clinical acceptability (ΔE_00_ > 1.8), while for GIC (F2) the value was near the threshold of acceptability (ΔE_00_ = 1.7). With increasing concentrations of ZnO-NPs, the ΔE_00_ values increased statistically significantly (*p* = 0.000). The difference was not statistically significant only between F_3_ and F_4_ group.

**Figure 4:**
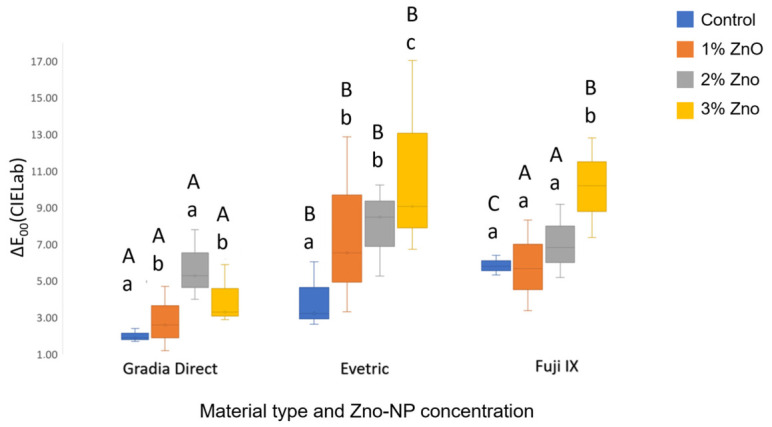
Changes in the color of the materials (ΔE_00_) after immersion in red wine. Same uppercase letters indicate no significant differences between different materials with the same ZnO-NP concentration (*p* > 0.05); same lowercase letters indicate no significant differences within one type of material with different ZnO-NP concentrations (*p* > 0.05). Uppercase letters above the bars indicate no statistically significant differences between different materials with the same ZnO-NPs concentration (*p* > 0.05). Lowercase letters indicate no statistically significant differences within one type of material across different ZnO-NP concentrations (*p* > 0.05). Different letters (either uppercase or lowercase) denote statistically significant differences (*p* < 0.05) between the corresponding groups. The labels “A”, “B”, etc., are assigned based on the values of the parameter. “A” is assigned to the material with the highest value. The next material is assigned the label “B” if the difference is statistically significant; otherwise, it is labeled “A.” The following material is compared with the previous one using the same principle. Lowercase letters within each material are assigned according to the same principle as uppercase letters.

Following immersion in red wine, all tested materials exhibited color changes exceeding the clinical acceptability threshold (ΔE_00_ > 1.8) (Figure 4).

Within all three material groups, statistically significant differences in ΔE_00_ values were observed, depending on the concentration of incorporated ZnO-NPs. As the percentage of incorporated ZnO-NPs increased, ΔE_00_ values also increased, except for G_4_ compared to the G3 group. Within the Gradia Direct groups, no statistically significant difference (*p* > 0.05) was observed only between G_1_ and G_2_ and between the G2 and G_4_ groups. In the Evetric groups, a statistically significant difference (*p* = 0.000) was observed between group E_1_ and the other groups (E_2_, E_3_, and E_4_) but no significant differences (*p* = 0.687) were found among groups E_2_, E_3_, and E_4_ mutually. Within the Fuji IX groups, statistically significant differences were noted among F_1_ and F_4_ (*p* = 0.005), F_2_ and F_4,_ (*p* = 0.005), F_3_ and F_4,_ (*p* = 0.005), while differences between F_1_ and F_2,_ (*p* = 0.675), F_1_ and F_3_ (*p* = 0.084) as well as between F_2_ and F_3_ were not significant (*p* = 0.280).

The results also indicate that there is a statistically significant difference in ΔE_00_ (*p* = 0.000) between the materials at the same concentration (%) of added ZnO-NPs after immersion in red wine. Specifically, significant differences were observed between Gradia Direct and Evetric, as well as between Gradia Direct and Fuji IX, across all ZnO-NPs concentrations. In contrast, the difference between Evetric and Fuji IX was statistically significant only at the 2% ZnO-NPs concentration (*p* = 0.000).

Notably, all RBC groups modified with ZnO-NPs exhibited ΔE_00_ values exceeding 1.8, including at the lowest concentration (1 wt%). This indicates that even minimal ZnO-NP incorporation can result in a color change that is perceptible to the human eye, which may have important clinical implications for esthetic restorations.

**Figure 5:**
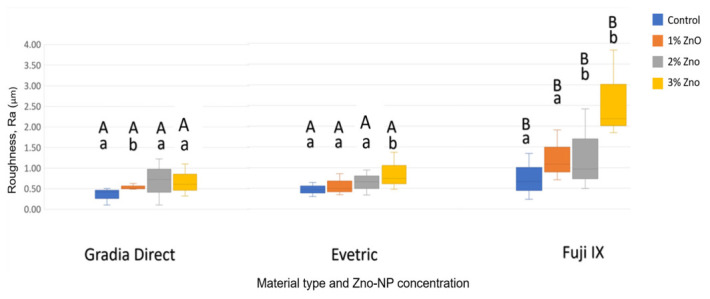
Surface roughness (Ra) of materials incorporated with ZnO-NPs. Same uppercase letters indicate no significant differences between different materials with the same ZnO-NP concentration (*p* > 0.05); the same lowercase letters indicate no significant differences within one type of material with different ZnO-NP concentrations (*p* > 0.05). Uppercase letters above the bars indicate no statistically significant differences between different materials with the same ZnO-NPs concentration (*p* > 0.05). Lowercase letters indicate no statistically significant differences within one type of material across different ZnO-NPs concentrations (*p* > 0.05). Different letters (either uppercase or lowercase) denote statistically significant differences (*p* < 0.05) between the corresponding groups. The labels “A”, “B”, etc., are assigned based on the values of the parameter. “A” is assigned to the material with the highest value. The next material is assigned the label “B” if the difference is statistically significant; otherwise, it is labeled “A.” The following material is compared with the previous one using the same principle. Lowercase letters within each material are assigned according to the same principle as uppercase letters.

The surface roughness measurement results are presented in Figure 5. For all groups, the surface roughness (Ra) was evaluated and compared to the clinically accepted surface roughness threshold for plaque retention, which is typically considered to be 0.2 μm. In this study, all tested groups exhibited Ra values above this threshold, indicating a higher risk for plaque accumulation. The highest surface roughness of pure commercial materials was measured for GIC (F1) (Ra = 0.92), and the lowest was for RBC Gradia Direct (G1) (Ra = 0.35). With the incorporation of ZnO-NPs, the surface roughness of all tested materials increased. No statistically significant difference (*p* > 0.05) was observed in surface roughness between the Gradia Direct and Evetric groups across all ZnO-NPs concentrations; however significant difference was found between the GIC groups and RBC groups.

Also within all materials, a significant difference in surface roughness was observed depending on the concentration of ZnO-NPs. Within the Gradia Direct groups, a statistically significant difference (*p* = 0.006) was observed between control group (G_1)_ and the other groups (G_2,_) but no significant differences were found among groups G_2_ (*p=* 0.393), G_3_, and G_4_ (*p =* 1.000) mutually. In the Evetric groups, a statistically significant difference (*p* = 0.006) was observed between control group E_1_ compared to E_4_ groups. Within the GIC groups, statistically significant differences were noted between the control group (F_1_) and groups F_3_ (*p* = 0.016) and F_4,_ (*p* = 0.000) as well as between F_2_ and F_4_ (*p* = 0.000)_,_ and between F_3_ and F_4_ (*p* = 0.016).

The obtained results, *p* values and effective sizes are additionally presented in Supplementary Tables S1–S3 in the Supplementary Materials.

## 4. Discussion

The null hypotheses were rejected as statistically significant differences were observed in the surface roughness, initial color properties, and color change among the tested materials. Consequently, the results clearly indicate that the incorporation of ZnO-NPs significantly modulates these parameters. While we acknowledge that manual mixing is a suboptimal method for achieving ideal nanoparticle dispersion—as evidenced by the agglomeration zones in our SEM micrographs—this limitation is not unique to the present study. Similar manual mixing techniques have been widely documented in the literature on dental materials, particularly in exploratory studies focusing on ZnO-NP incorporation. Such approaches are considered valid for establishing baseline effects, assessing feasibility, and evaluating initial property alterations [22,32,33,34]. Moreover, manual mixing closely mimics the pragmatic challenges faced during clinical or chairside procedures, where industrial-scale dispersion technologies are unfeasible.

In this study, two different commercial composite materials were tested: a microhybrid UDMA-based and a nanohybrid BisGMA-based, along with one conventional glass ionomer cement, Fuji IX, and their modifications with the addition of 1 wt%, 2 wt%, or 3 wt% ZnO-NPs. Shade A3 was selected as it is one of the most frequently utilized shades in daily clinical practice. In the present study, a 24 h continuous solution immersion protocol was utilized. According to the mathematical model established by Ertaş et al. [35] the average time required to consume one drink is estimated at 15 min, meaning that a 24 h in vitro immersion corresponds to 96 clinical consumption episodes. Consequently, assuming a regular dietary intake of one glass of red wine per day, this 24 h protocol effectively simulates approximately three months of clinical exposure. For the threshold of clinical acceptability of color change, a value of ΔE_00_ = 1.8 was used according to Paravina et al. [37].

The initial optical properties of restorative materials depend on the material composition, the degree of polymerization, and the surface treatment of the restoration. Although all commercial materials were of shade A3, their measured initial L*, a*, and b* values differed from one another. GIC had the highest brightness and lower values for the a* and b* parameters compared to both RBCs. The results of this study showed that the incorporation of ZnO-NPs led to a change in the initial color of all tested materials. By analyzing the L*, a*, and b* values, it was observed that with the addition of ZnO-NPs, all three tested materials became brighter; there was an increase in L* values. The lowest change in L* values was observed in GIC (F) groups, indicating that the material with the initially highest L* values changes brightness the least. This result can be considered expected, given that ZnO-NPs are white, and it is also known that ZnO is used to achieve white color in painting [38]. Simultaneously, a decrease in a* values was observed, suggesting that ZnO-NPs partially neutralize the red component of the matrix. The decrease in yellow tones (b* values) in RBCs and the increase in GICs indicate that the chemical composition of the material influences the eventual interaction of ZnO-NPs with the polymer matrix.

The analysis of the ΔE_00_ values revealed that even a 1 wt% incorporation of ZnO-NPs into RBCs induces a color change exceeding the clinical acceptability threshold (ΔE_00_ > 1.8), whereas the alteration for the GIC remains close to the limit (ΔE_00_ = 1.7). The obtained result indicates that even small concentrations of ZnO-NPs can significantly change the optical properties of restorative materials. In general, the magnitude of color change was proportional to the concentration of incorporated ZnO-NPs. However, the GIC group exhibited a plateau, as concentrations exceeding 2 wt% did not induce a further increase in ΔE_00_. This phenomenon suggests a potential saturation of the material’s optical properties.

Data on the effects of added ZnO-NPs on the color of RBCs and GIC restorative materials is lacking in the literature. However, in a study by Rudolf et al., the addition of ZnO-NPs to denture base material also resulted in a brighter material [39]. A similar tendency was observed in the study by Szerszeń et al., where color changes of PMMA increased with increasing ZnO-NPs concentration, which was attributed to the increased dispersion and interaction of the nanoparticles with light within the material [17].

These color changes caused by the introduction of metal-oxide nanoparticles are anticipated and can be attributed to the mismatch between the refractive indices of the nanoparticles and the polymer matrix. This phenomenon directly modulates light propagation through the material, along with specific absorption and reflection pathways, both of which are well-documented as driving factors behind the optical transformations in nanomodified polymers [40]. Since the nanoparticles are smaller than the wavelength of visible light, their homogeneous dispersion enables precise control over light absorption and scattering. Conversely, the presence of larger agglomerates induces perceptible aesthetic changes, which critically impacts the clinical appearance of the restoration [41].

The results carry significant implications for the potential integration of ZnO-NPs into commercial materials. The observed impact on color—particularly in darker shades and those with a pronounced b* component—could be mitigated through the addition of appropriate pigments. Numerous studies have shown that RBCs and GIC undergo color changes after exposure to staining solutions. According to the existing literature, red wine has demonstrated the highest staining potential for composite materials [19,21,42] due to the presence of anthocyanins [21]; therefore, it was selected as the staining medium for the present study.

The obtained results showed that, after immersion in red wine, the color change was generally lowest for Gradia Direct, both for the pure material and for all added concentrations of ZnO-NPs. Pure Evetric showed slightly less staining compared to Fuji IX, although the difference was not statistically significant. However, with the addition of ZnO-NPs, the ΔE_00_ values for Evetric were higher even compared to Fuji IX. Regarding the pure commercial materials, the obtained results are in accordance with previous studies. Bagheri et al. [43] and Clarence and Chakravarthy [20] also found that GIC has a greater color change than RBCs. The greater staining of GICs is attributed to their hydrophilic nature, higher water sorption, and greater porosity, which allows for easier pigment penetration and intrinsic discoloration. They are also less resistant to degradation in an acidic environment, such as wine, which leads to surface erosion and pigment retention. Arocha et al. [42] and Dervisevic et al. [44] also found less staining in UDMA-based composites (like Gradia Direct) compared to BisGMA-based composites (like Evetric) in their research. This result is explained by the fact that the hydrophilic hydroxyl groups of the BisGMA monomer led to higher water sorption compared to the aliphatic UDMA chain, which can result in greater pigment penetration and hydrolytic degradation. Although generally adding ZnO-NPs led to significantly greater staining of the material, the color change was not always higher with increased concentrations of ZnO-NPs. In the Evetric composite, even the lowest added concentration of ZnO-NPs led to an increase in ΔE_00_ values by double compared to the pure material (ΔE_00_ = 8.0 compared to ΔE_00_ = 4.1). In contrast, the addition of 1 wt% ZnO-NPs to Fuji IX did not lead to increased staining compared to the pure material. This finding suggests that, in addition to the material type and the amount of incorporated ZnO-NPs, other factors may play a role in staining, and synergistic or antagonistic effects may occur. BisGMA resin is more viscous and contains more hydroxyl groups, which can facilitate the non-uniform dispersion of ZnO-NPs and the formation of micro-aggregates that absorb light and increase ΔE_00_. Additionally, the ZnO-NPs themselves reflect and scatter light, which also changes color parameters alongside the adsorbed pigments. Furthermore, the inhomogeneous distribution and aggregation of added particles, which are observed in SEM micrographs, can significantly affect the color.

There is not much data in the literature regarding the impact of ZnO-NPs on the color and color stability of RBC, and especially not for GIC. In a study by Dias et al. [22], it was also shown that modifying the composite with 2 wt% ZnO-NPs leads to greater color change after immersion in coffee compared to the pure, unmodified RBC.

The fact that ΔE_00_ values exceeded the clinical acceptability threshold of 1.8 in all modified RBC groups, even at a 1 wt% ZnO-NP concentration, suggests that both clinicians and material manufacturers must carefully consider the trade-off between the desired antimicrobial effects and potential aesthetic compromises when selecting nanoparticle concentrations for aesthetic restorations.

According to literature data, better-polished, smoother surfaces of restorative materials also have lower surface energy and fewer retention zones, making them less susceptible to dental plaque adhesion [45]. However, literature data on the influence of surface roughness on staining are contradictory. Some studies suggest that rougher surfaces, due to greater adsorption and absorption of colored molecules from food and drinks, are more prone to discoloration [25], while others indicate that staining is not necessarily directly proportional to the surface roughness of the material [21,44,46].

In this study, significantly higher surface roughness was observed in GIC compared to both RBCs, while the difference between Gradia Direct and Evetric composites was not statistically significant, although slightly higher values were recorded for Evetric. The obtained result can be attributed to differences in the chemical composition and microstructure of the materials, specifically the bonding mechanism between the filler and the matrix. The greater surface roughness of GIC may be a consequence of the presence of large glass particles that are less stably integrated into the matrix and detach from it more easily, while composites generally have more homogeneous, smaller, and silanized filler particles, resulting in a smoother surface [47,48].

The incorporation of ZnO-NPs promoted an elevation in surface roughness across all tested materials. Generally, a trend was observed where a higher particle content resulted in greater roughness, although this difference was not statistically significant in all cases. While the antimicrobial potential of incorporated ZnO-NPs has been widely investigated in the literature, there are limited data on their direct impact on the surface roughness of RBCs and GICs.

Contrary to the findings of the present research, Jowkar et al. [26] reported that adding an optimal amount (5 wt%) of conventional and mesoporous ZnO-NPs can reduce the surface roughness of resin-modified GIC, whereas a higher concentration (7 wt%) results in a rougher surface due to particle agglomeration. Similarly, Jowkar et al. [26] and Agustantina et al. [27] observed that incorporation of ZnO-NPs decreased the surface roughness of GIC. This outcome was attributed to a reduction in the average particle size and the capacity of uniformly distributed nanoparticles to fill surface micro-irregularities. To date, no data have been found in the literature regarding composite materials intended for direct restoration. However, Mostafa & Sadoon [49] investigated this impact on 3D-printed PMMA, demonstrating that the incorporation of nanoparticles reduces surface roughness.

The increase in surface roughness observed in this study can be attributed to the spherical shape of the ZnO-NPs, which exhibit a greater tendency to agglomerate compared to rod-like nanoparticles [50]. It is expected that a homogeneous dispersion of nanoparticles could reduce surface roughness, whereas their agglomeration promotes an increase in this parameter.

While several studies have reported that incorporating ZnO-NPs reduces surface roughness, our findings, along with those of other researchers, indicate an increase in Ra values. This discrepancy may be attributed to several factors. First, variations in ZnO-NP concentration can influence particle aggregation; higher concentrations may promote agglomeration, leading to increased surface irregularities. Second, differences in particle size and the effectiveness of dispersion techniques play a crucial role: smaller or well-dispersed nanoparticles may fill resin matrix gaps more effectively, smoothing the surface, whereas poor dispersion results in rougher textures. Finally, the composition of the restorative material matrix itself, alongside differences in sample preparation and finishing protocols, may contribute to inconsistent outcomes across studies. These variables highlight the need for standardized methodologies when evaluating the impact of nanoparticle incorporation on surface properties.

When observing the surface roughness results in the context of color change after immersion, it is evident that an increase in surface roughness does not consistently predict greater discoloration for all materials. Although the GIC exhibited significantly higher roughness than Evetric both initially and after ZnO-NP incorporation, its staining after immersion in red wine was not proportional. This outcome aligns with the research by Janson et al. [21] and Dervisevic et al. [44], who also found no direct correlation between increased surface roughness and the intensity of color change. In contrast, Aydın et al. [25] reported that higher surface roughness contributes to greater color change due to enhanced adsorption and retention of staining molecules. While all groups showed increased surface roughness (Ra) and discoloration (ΔE_00_) with ZnO-NP incorporation, our results revealed no direct, proportional correlation between these two properties, indicating that factors beyond surface texture—such as material composition, chemical structure, polymer matrix, and fillers—heavily influence stain susceptibility.

It is noteworthy that Ra values exceeding 0.2 μm are closely linked to increased plaque retention and biofilm formation, potentially compromising oral hygiene and restoration longevity [51]. In the present study, Ra values remained above this threshold across all evaluated groups, emphasizing the importance of achieving antimicrobial benefits without sacrificing surface quality.

Recent studies by Jowkar et al. [26] and Malekhoseini et al. [29] have significantly advanced the understanding of ZnO-NP incorporation in resin-modified GICs, particularly regarding mechanical and antimicrobial properties. However, their focus remained largely confined to a single material type and did not address key aesthetic properties, such as color stability, in depth. In contrast, the present study systematically examined not only surface roughness but also initial color, color change after exposure to a strong staining agent (red wine), utilizing the clinical acceptability threshold (ΔE_00_ > 1.8) to interpret the optical results. To our knowledge, this is the first study to apply these criteria in a direct comparison of these material classes under simulated clinical conditions. These findings thus add a novel and clinically relevant dimension to the existing literature by demonstrating the interplay between nanoparticle concentration, optical properties, and surface characteristics under realistic challenges. Consequently, these insights may guide material selection and future formulation strategies for optimizing both the antimicrobial potential and the aesthetic longevity of restorative materials.

The obtained results are important for understanding the factors that influence color, color stability and surface roughness, which are essential for optimizing material selection and achieving favorable clinical outcomes. Future studies should include a wider range of concentrations to optimize the amount of added nanoparticles, different particle shapes, consideration of the possible application of silanized particles and better homogenization of the material to reduce the possibility of agglomeration of the added nanoparticles.

The observed optical changes, including color shifts and reduced translucency, can be attributed to several underlying mechanisms. The addition of ZnO-NPs introduces a refractive index mismatch between the inorganic fillers and the organic resin matrix. This difference causes increased light scattering at the nanoparticle–matrix interfaces, leading to reduced translucency and perceptible color alterations. Furthermore, agglomeration of nanoparticles can create larger, irregular domains that scatter light more intensely, exacerbating these optical effects. The degree of scattering and color change is therefore dependent not only on the concentration of nanoparticles but also on their dispersion and compatibility with the matrix.

The clinical significance of the observed changes in color (ΔE_00_) and surface roughness (Ra) should be emphasized. A ΔE_00_ value greater than 1.8 is widely accepted as the threshold for clinically unacceptable color change in restorative dentistry [37]. In this study, all RBC groups modified with ZnO-NPs exceeded this threshold, even at the lowest concentration, suggesting that such modifications may compromise the aesthetic longevity of restorations. Regarding surface roughness, an Ra value exceeding 0.2 μm is considered to increase the risk of bacterial plaque retention and subsequent development of secondary caries [51]. All tested groups in this study displayed Ra values above this threshold, indicating a potential risk for biofilm accumulation and adverse clinical outcomes.

Therefore, although the literature indicates that the incorporation of ZnO-NPs can offer antimicrobial potential, this modification could negatively affect both the aesthetic appearance and the long-term maintenance of restorations. Clinicians and manufacturers should carefully weigh these risks against potential benefits when considering the use of ZnO-NP-modified materials. Further optimization of nanoparticle concentration and dispersion protocols is required before clinical adoption.

The agglomeration of ZnO-NPs may play a central role in influencing all measured properties. Agglomerated nanoparticles can create surface irregularities, increase roughness, and result in non-homogeneous color distribution, thereby leading to perceptible color changes. Furthermore, these agglomerates may act as stress concentrators or weak points within the material, potentially compromising its mechanical properties and durability. These findings underscore the importance of employing advanced dispersion techniques and conducting thorough characterization of nanoparticle distribution in restorative materials to minimize agglomeration and accurately assess the effects of nanoparticle incorporation.

A notable methodological limitation of this study was the use of manual mixing for incorporating the ZnO-NPs into the restorative materials. As confirmed by our SEM analysis, this approach resulted in visible agglomeration rather than a uniform nanoparticle dispersion. These agglomerates can act as light-scattering centers and create surface irregularities, potentially leading to more pronounced changes in both color and surface roughness than would be expected with an optimal nanoparticle distribution. Thus, the observed effects may represent an upper bound, and improvements in mixing techniques, such as mechanical stirring or sonication, could reduce agglomeration and yield more consistent results. Future studies should incorporate advanced dispersion methods and quantitative analyses of nanoparticle distribution to better isolate the true impact of ZnO-NPs on the properties of these materials.

In addition, future research should investigate a broader range of ZnO-NP concentrations to delineate the optimal balance between antimicrobial efficacy, color stability, and mechanical properties. Furthermore, surface modification of the ZnO-NPs, such as functionalization via silanization, may enhance interfacial compatibility with the polymer matrix, mitigate agglomeration, and potentially improve both aesthetic and functional outcomes. The use of advanced dispersion techniques (e.g., mechanical stirring, ultrasonication, or in situ polymerization) should also be explored to achieve more homogeneous nanoparticle distribution within the restorative materials. These strategies could effectively rectify the current limitations associated with manual mixing, thereby paving the way for the development of clinically superior nanomodified dental materials.

### Limitations

Several limitations inherent to this study warrant consideration regarding the clinical applicability of the results. First, the in vitro nature of the design cannot fully encapsulate the complex, dynamic thermal and biochemical conditions encountered in vivo. Second, the primary methodological constraint pertains to the manual mixing technique employed for ZnO-NP incorporation. This approach yielded localized nanoparticle agglomeration rather than a homogeneous dispersion. These clusters likely functioned as light-scattering centers and exacerbated surface irregularities, meaning the recorded optical and textural shifts might represent an upper bound rather than the baseline behavior of well-dispersed systems.

Additionally, the 24 h immersion protocol lacks comprehensive aging simulations, such as thermocycling or extended storage, which are essential to mimic the long-term chemical and mechanical stresses of the oral cavity. This temporal limitation is further compounded by the fact that surface roughness was quantified solely prior to red wine exposure. Because acidic staining media can induce surface degradation and erosion, post-staining topographic characterization remains necessary to fully evaluate structural durability.

To address these limitations, future research should employ advanced dispersion methodologies, such as mechanical stirring, sonication, or in situ polymerization, alongside a comprehensive characterization of nanoparticle size and morphology via TEM or DLS. Furthermore, long-term evaluation protocols incorporating extended aging, post-staining roughness assessments, and stringent statistical corrections are required to fully elucidate the clinical performance of these nanomodified restorative materials.

## 5. Conclusions

In conclusion, incorporating 1–3 wt% ZnO-NPs into restorative dental materials significantly influenced both color stability and surface roughness, with higher concentrations leading to more pronounced alteration. These findings suggest that ZnO-NP modification can impact the clinical performance of dental materials; therefore, optimal concentrations must be carefully selected to balance the desired functional benefits with the preservation of aesthetic and physical properties. However, given the absence of antimicrobial testing and long-term evaluation, these results should be considered preliminary and cannot yet be directly translated into clinical practice. Further research is required to assess the long-term durability of these materials over extended periods before clinical recommendations can be established. To our knowledge, this is the first study to directly compare the impact of ZnO-NP incorporation on both resin-based composites and conventional glass-ionomer cements, highlighting important differences and similarities that have not been previously addressed in the literature.

## Figures and Tables

**Table 1:** Materials investigated in this study.

Group	Material
G_1_	Gradia Direct (Control, 0% ZnO-NPs)
E_1_	Evetric (Control, 0% ZnO-NPs)
F_1_	Fuji IX (Control, 0% ZnO-NPs)
G_2_–G_4_	Gradia Direct + 1%, 2% or 3% ZnO-NPs
E_2_–E_4_	Evetric +1%, 2% or 3% ZnO-NPs
F_2_–F_4_	Fuji IX + 1%, 2% or 3% ZnO-NPs

**Table 2:** X-ray diffraction data and calculated crystallite sizes for the synthesized ZnO-NPs calculated from diffraction peaks with *hkl* Miller indices.

Peak No./*hkl*	β_measured_ (Rad)	β_instrumental_ (Rad)	β (Rad)	2θ (°)	*D* (nm)
1/(100)	0.00517	0.00105	0.00506	31.68	28.2
2/(002)	0.00551	0.00105	0.00541	34.32	26.5
3/(101)	0.00590	0.00105	0.00581	36.12	24.8

**Table 3:** Mean values of L*, a*, and b* color coordinates and their standard deviations for the tested materials.

Material	L*	a*	b*
Gradia Direct (G_1_) − control	66.5 ± 0.8	3.4 ± 0.2	17.5 ± 0.4
Gradia Direct + 1% ZnO-NPs (G_2_)	74.8 ± 0.3	3.0 ± 0.1	13.2 ± 0.4
Gradia Direct + 2% ZnO-NPs (G_3_)	78.9 ± 0.4	2.5 ± 0.3	11.4 ± 0.2
Gradia Direct + 3% ZnO-NPs (G_4_)	80.9 ± 0.5	2.0 ± 0.4	10.8 ± 0.6
Evetric (E_1_) − control	66.4 ± 0.5	1.9 ± 0.1	15.0 ± 0.5
Evetric + 1% ZnO-NPs (E_2_)	74.7 ± 0.4	0.7 ± 0.2	10.2 ± 0.3
Evetric + 2% ZnO-NPs (E_3_)	80.0 ± 0.4	0.7 ± 0.1	8.1 ± 0.3
Evetric + 3% ZnO-NPs (E_4_)	81.5 ± 0.3	0.9 ± 0.1	7.9 ± 0.4
FUJI IX (F_1_) − control	81.1 ± 0.7	1.2 ± 0.1	7.9 ± 1.1
FUJI IX + 1% ZnO-NPs (F_2_)	83.2 ± 1.4	1.1 ± 0.2	6.4 ± 1.5
FUJI IX + 2% ZnO-NPs (F_3_)	84.9 ± 1.3	−1.5 ± 0.3	9.9 ± 1.2
FUJI IX + 3% ZnO-NPs (F_4_)	84.8 ± 1.9	−1.3 ± 0.4	9.0 ± 1.4

## Data Availability

The data presented in this study are available in: Zenodo, at https://doi.org/10.5281/zenodo.19930964.

## Supplementary Materials

The following supporting information can be downloaded at: https://www.mdpi.com/article/10.3390/jfb17070343/s1, Table S1: Mean color changes in the initial color of the materials (ΔE_00_) after the addition of different concentrations of ZnO-NPs; Table S2: Changes in the color of the materials (ΔE_00_) after immersion in red wine; Table S3: Comparative Surface Roughness of the materials (Ra) with ZnO-NPs incorporation.
