# Supplementary material for: Influence of ZnO Nanoparticles on the Color and Surface Roughness of Composite and Glass-Ionomer Materials
Source: J Funct Biomater. 2026 Jul 15;17(7):343. doi: 10.3390/jfb17070343 (PMC13413418; doi:10.3390/jfb17070343)
Supplement: Supplementary file 1 [file jfb-17-00343-s001.zip › jfb-4398907-supplementary.pdf]

Supporting Information

Influence of ZnO Nanoparticles on the Color and Surface Roughness of Composite and Glass-Ionomer Materials

Sanja Ilić [1], Neda Ninkovic[2], Branislav Sredanovic[3], Goran Vučić [4], Ljiljana Božić [5], Ljubica Škrbić [1], Jovana Kuzmanovic Pficer [6] and Dragica Manojlovic [2,*]

[1] Dental Clinic, Faculty of Medicine, University of Banja Luka, Bulevar vojvode Petra Bojovića 1a, 78000 Banja Luka, Bosnia and Herzegovina; sanja.ilic@med.unibl.org (S.I.); ljubica.skrbic@med.unibl.org (L.Š.)
[2] Department of Restorative Odontology and Endodontics, School of Dental Medicine, University of Belgrade, Rankeova 4, 11000 Belgrade, Serbia; neda.nikolic@stomf.bg.ac.rs
[3] Department for Production and Computer Aided Technologies, Faculty of Mechanical Engineering, University of Banja Luka, Bulevar vojvode Stepe Stepanovića 71, 78000 Banja Luka, Bosnia and Herzegovina; branislav.sredanovic@mf.unibl.org
[4] Faculty of Technology, University of Banja Luka, Bulevar vojvode Stepe Stepanovića 73, 78000 Banja Luka, Bosnia and Herzegovina; goran.vucic@tf.unibl.org
[5] Department of microbiology and immunology, Faculty of Medicine, University of Banja Luka, Save Mrkalja 14, 78000 Banja Luka, Bosnia and Herzegovina; ljiljana.bozic@med.unibl.org
[6] Department for Medical Statistics and Informatics, School of Dental Medicine, University of Belgrade, Dr. Subotica 1, 11000 Belgrade, Serbia; jovana.kuzmanovic@stomf.bg.ac.rs
* Correspondence: dragica.manojlovic@stomf.bg.ac.rs; Tel.: +381-112685288

Table S1. Mean color changes in the initial color of the materials (ΔE_{00}) after the addition of different concentrations of ZnO-NPs. Same uppercase letters indicate no significant differences between different materials with the same ZnO-NPs concentration ($p > 0.05$); same lowercase letters indicate no significant differences within one type of material with different ZnO-NPs concentration ($p > 0.05$).

	**Material**				
**% ZnO**	Gradia Direct (ΔE_{00})	Evetric (ΔE_{00})	Fuji IX (ΔE_{00})	P value	η^2
**1**	$4.35 \pm 0.17^{A,a}$	$4.85 \pm 0.12^{A,a}$	$1.73 \pm 1.23^{A,a}$	**0.000**	0.401
**2**	$6.33 \pm 0.17^{A,b}$	$7.31 \pm 0.19^{B,b}$	$4.93 \pm 0.56^{B,b}$	**0.000**	0.892
**3**	$7.24 \pm 0.222^{B,c}$	$4.93 \pm 0.57^{C,c}$	$4.65 \pm 0.41^{C,b}$	**0.000**	0.959

Table S2. Changes in the color of the materials (ΔE_{00}) after immersion in red wine. Same uppercase letters indicate no significant differences between different materials with the same ZnO-NPs

concentration ($p > 0.05$); same lowercase letters indicate no significant differences within one type of material with different ZnO-NPs concentration ($p > 0.05$).

	Material				
**% ZnO**	Gradia Direct (ΔE_{00})	Evetric (ΔE_{00})	Fuji IX (ΔE_{00})	P value	η^2
**Control**	$1.97 \pm 0.28^{A,a}$	$4.09 \pm 1.01^{B,a}$	$6.14 \pm 0.29^{C,a}$	**0.000**	0.804
**1**	$2.82 \pm 1.24^{A,b}$	$7.97 \pm 3.57^{B,b}$	$6.11 \pm 1.33^{A,a}$	**0.002**	0.483
**2**	$5.41 \pm 1.06^{A,a}$	$8.73 \pm 1.78^{B,b}$	$6.96 \pm 0.96^{A,a}$	**0.000**	0.537
**3**	$3.65 \pm 0.86^{A,b}$	$10.56 \pm 2.92^{B,c}$	$9.77 \pm 1.91^{B,b}$	**0.000**	0.646

Table S3. Comparative Surface Roughness of the materials (Ra) with ZnO-NPs incorporation. Same uppercase letters indicate no significant differences between different materials with the same ZnO-NPs concentration ($p > 0.05$); same lowercase letters indicate no significant differences within one type of material with different ZnO-NPs concentration ($p > 0.05$).

	Material				
**% ZnO**	Gradia Direct (μm)	Evetric (μm)	Fuji IX (μm)	P value	η^2
**Control**	$0.35 \pm 0.15^{A,a}$	$0.47 \pm 0.09^{A,a}$	$0.92 \pm 0.24^{B,a}$	**0.006**	0.687
**1**	$0.52 \pm 0.03^{A,b}$	$0.54 \pm 0.15^{A,a}$	$1.16 \pm 0.32^{B,a}$	**0.000**	0.617
**2**	$0.62 \pm 0.36^{A,a}$	$0.67 \pm 0.21^{A,a}$	$1.56 \pm 0.49^{B,b}$	**0.000**	0.600
**3**	$0.60 \pm 0.25^{A,a}$	$0.77 \pm 0.26^{A,b}$	$2.37 \pm 0.62^{B,b}$	**0.000**	0.680
